# Leveraging Stakeholder Engagement for Adolescent School Journeys in Malawi: An Exploration of Road Safety and Air Pollution Interventions

**DOI:** 10.3390/ijerph22050758

**Published:** 2025-05-12

**Authors:** Dennis Mazingi, Prasanthi Puvanachandra, Alejandra Piragauta, Bosco Exson Chinkonda, Monica Nzanga, Linda Chokotho, Margaret Mary Peden

**Affiliations:** 1 The George Institute for Global Health UK, London W12 7RZ, UKapiragauta@georgeinstitute.org.uk (A.P.); mpeden@georgeinstitute.org.uk (M.M.P.); 2 School of Public Health, Imperial College, London W12 0BZ, UK; 3 School of Population Health, University of New South Wales, Sydney 2052, Australia; lindachokotho@gmail.com; 4 Department of Community and Environmental Health, Kamuzu University of Health Sciences, Chichiri, Blantyre 3, Malawi; boscochinkonda@gmail.com (B.E.C.); monicanzanga0@gmail.com (M.N.)

**Keywords:** road safety, air pollution, school journeys, stakeholder engagement, Malawi

## Abstract

Road traffic injuries (RTIs) and air pollution present dual burdens that disproportionately affect school-going children in low-income urban settings like Malawi. Despite availability of evidence-based interventions, their implementation often overlooks local contexts and perspectives. This study aimed to elicit stakeholder input on interventions addressing RTIs and air pollution exposure among children in urban Blantyre through stakeholder engagement. It used a mixed method Delphi technique combining expert consultations with community focus groups to achieve consensus on interventions. Successive rounds of prioritization and qualitative discussions explored contextual barriers and facilitators to implementation. Stakeholders identified 40 interventions, 23 for road safety and 17 for air pollution. Measures prioritized by experts included speed limit enforcement, pedestrian infrastructure improvements, and emission controls. Contextual barriers identified by experts and the community included socio-political and financial constraints. Community perspectives emphasized behavioral interventions, while experts highlighted systemic and legislative changes. The study underscored the value of combining expert and community perspectives to design context-sensitive interventions. Synergies between road safety and air pollution interventions offer opportunities for dual benefits but require careful adaptation to urban Malawi’s realities. This study provides a model for participatory design in low-income settings, emphasizing stakeholder engagement for tailored solutions.

## 1. Introduction

Globally, around 350,000 children and adolescents die each year from road traffic collisions and urban air pollution [1], with road traffic collisions being a leading cause of death among 5-to-19-year-olds [2]. Ninety-three per cent of these child and adolescent traffic fatalities occur in low- and middle-income countries [2], where they also suffer from the harmful effects of traffic-related air pollutants (TRAPs) [3], leading to stunted growth, cognitive delays, and chronic respiratory issues [4]. The common risk factors associated with road traffic injuries and air pollution often lead to simultaneous exposure for these children, creating a dual burden of disease [5], with the potential for these factors to interact with one another [6,7].

In Malawi, a low-income country in southern Africa, the road traffic death rate was 20 per 100,000 population in 2021 [2] and the level of fine particulate matter (PM_2.5_) was more than double the World Health Organization safe threshold of 10 µg/m^3^ [8]. Pedestrians account for half of the traffic fatalities, and RTIs among children and adolescents have risen steeply in the past decade, making up 53.8% of hospital admissions [9]. Air pollution is linked to chronic respiratory symptoms in up to 16.7% of children, abnormal spirometry results in 13.0%, and exposure levels exceeding WHO guidelines in 50% to 68% of cases [10]. Many children in Malawi walk to school, and many schools are located along major highways, increasing their exposure to high-speed traffic and high levels of TRAPs [11].

Several effective, evidence-based interventions exist to reduce pedestrian injuries [12,13,14] and improve air quality [15,16]. However, these have often been implemented separately, despite the need for an integrated approach that simultaneously addresses road safety and air pollution. This is particularly relevant in the African context, where local adaptation of interventions in Tanzania [17] and Ghana [18], for example, have been crucial to their success. Despite the availability of well-tested interventions, there are concerns about their implementation, particularly in Africa, where they may be applied without necessary modifications to align with local cultural, socio-economic, and environmental conditions [19]. In addition, the unique challenges in the African context may make implementation particularly challenging, requiring a multisectoral, collaborative approach. Engaging local communities, stakeholders, and governments in the development and execution of these interventions is essential for ensuring their success.

Recent work by Jessani et al. supports this view [20]. Deliberate engagement processes between researchers and decision-makers facilitate the development of contextually relevant interventions and require capacity building, authentic relationship development, and agile multi-sectoral engagement, not as a one-time consultation but as an ongoing process that bridges the gap between evidence production and practical implementation.

To the best of our knowledge, there are no published studies that have addressed both road safety and air pollution among school-going children in Africa.

This study aimed to identify targeted interventions for improving road safety and air quality for school children in urban Blantyre, Malawi. Using a modified Delphi methodology, the researchers engaged stakeholders to gather diverse perspectives and achieve consensus on effective strategies. By combining local knowledge with expert insights, the researchers hope to provide insights for a future comprehensive intervention package that enhances children’s safety on their journeys to school while contributing to broader discussions on sustainable transport and public health.

Developing effective and sustainable interventions requires a comprehensive understanding from various perspectives. Experts can propose evidence-based solutions, whereas the community perspectives highlight the nuances that may be overlooked by experts. Coproduction of interventions “maximises the likelihood of intervention effectiveness by improving the fit with the target group’s perceived needs and thus acceptability; practicality; evaluability” [21].

The aim of the study was as follows:

1. To identify and seek consensus on potential interventions for the prevention of road crashes and air pollution on school children’s journeys to school in urban Blantyre, Malawi.
2. To explore contextual barriers and facilitators to implementation of preventative interventions in urban Blantyre.

## 2. Materials and Methods

This study utilized a mixed method Delphi approach/qualitative study. The stakeholder engagement was initially based on a Delphi methodology, which is a systematic and interactive approach for achieving consensus based on structured, iterative communication with a panel of experts [22,23]. It allows for consensus building among the experts, making it suitable for contexts where individual judgements need to be combined into a collective output. However, the timeframe of the overall study and geographic context of the issue, combined with the need to incorporate diverse expert and community perspectives, led to considerable modification of the classical Delphi methodology to suit the unique circumstances that were encountered in Blantyre [24]. Therefore, the researchers modified the Delphi technique by adding a qualitative exploration of identified interventions and by utilizing a “rapid-Delphi” with successive rounds on the same day. This study was conducted between February and July 2023.

The modified methodology employed was as follows:

1. Identification and recruitment of local and international stakeholders.
2. Successive consensus rounds.
  (a) First-round identification of potential interventions from experts.
    - Feeding these interventions to community members (teachers and parents from urban schools in Blantyre) to understand barriers and facilitators.
  (b) Second-round ranking of interventions by experts.
    - Focus group discussions among experts.
  (c) Third-round ranking of interventions by experts.
    - Mapping of interventions onto relevant evidence-based frameworks.
3. Reduction of intervention list based on evidence and response analysis.

### 2.1. Identification and Recruitment

Experts in road safety and air pollution were identified by the team through a rapid review of the recent literature and snowball sampling through literature reference lists and bibliographies, as well as from individuals known to the authors. Experts were drawn from medical and educational institutions, non-governmental organizations, and policymakers and included a mix of pediatric, surgical, and respiratory medical professionals from national and international health institutions; researchers; national and international road safety experts; environmental experts; and local non-governmental organizations (NGOs) related to road safety, child health, environmental safety, and respiratory diseases. All respondents were invited to participate in the modified Delphi study via email or, where appropriate, a physical letter. This formal invitation included information about the study and a consent declaration; only invitees who replied and consented to take part were included.

Inclusion criteria for participation included the ability to speak English and a willingness to participate. For each participant type, further criteria were applied, as shown in Table 1.

### 2.2. Successive Consensus Rounds

The first round employed a web survey (Table S1). An individualized link was sent to the initial list of identified participants. Participants were asked to identify potential interventions for improving road safety and air pollution on school journeys for children in urban Blantyre. The researchers also asked experts to identify barriers and facilitators for implementing each intervention identified. The survey has been included in the Supplementary Material.

An in-person focus group discussion was then conducted with community members (parents and teachers of Chirimba and Kanjedza schools in Blantyre, Malawi, pre-selected from a previous study [25]). They were presented with the identified interventions from Round 1 to understand their perspectives on the barriers and facilitators for implementing the different interventions identified by experts.

The second round of this expert engagement was conducted through a workshop with local experts in Blantyre and an online workshop with international experts to prioritize road safety and air pollution interventions. During these workshops, each group of experts was presented with the list of interventions from Round 1 through a second web-based survey (Table S2). During Round 2, experts were asked to rank the interventions according to implementation priority. This was followed immediately by an hour-long expert focus group discussion where they discussed their prioritization and reasons (Table S4). Experts were also provided with synthesized community perspectives of barriers and facilitators from the earlier community focus group discussion (Table S5) and asked to discuss these and consider them for the next round. In the third round, experts were asked to re-rank the Interventions using insights gained from their focus group and the community perspectives (Table S3). The third online survey was held on the same day to maintain engagement with the process.

Between successive rounds, the researchers tried to improve response rates by directly re-contacting and reminding invited participants (via multiple contact methods), contacting colleagues of invitees and physical visits.

### 2.3. Reduction of Intervention List

Identified interventions were mapped onto evidence-based interventions in each category. Road safety interventions were aligned with the Save LIVES package of 22 evidence-based actions [12]. Air pollution interventions were mapped onto a modified framework (Burns et al.) based on the published literature on reducing airborne particulate matter/pollution [15,16]. For both road safety and air quality, interventions that were not supported by evidence of effectiveness were removed from the list, as their implementation could not be recommended. The remaining interventions were then renamed to align with their evidence-based counterparts where the meaning was similar.

### 2.4. Statistical Analysis

Data were exported into Microsoft Excel © (Version 2502). Respondent characteristics were summarized using descriptive statistics. The ranking process during Rounds 2 and 3 employed a weighted scoring system to prioritize interventions. During each Delphi round, experts ranked interventions in order of perceived priority within each intervention domain (e.g., enforcement, legislation and policy, speed control, infrastructure, etc.). Each ranking position was assigned a descending weight based on the number of interventions in that domain group; first-rank positions received the highest weight (e.g., a weight of 4 where there are 4 interventions within that domain), second-rank positions received a lower weight, third-rank positions received a descending rate, and so on (to the last ranked positions receiving a weight of 1). The total weighted score for each intervention was calculated by multiplying the number of participant votes at each rank by its corresponding weight and summing these products. After calculating the total weighted score for each intervention, the researchers determined the overall rank within its category (e.g., enforcement, infrastructure). The final normalized score was then calculated by dividing the total weighted score by the number of options in each category to allow for comparison across categories with different numbers of interventions using the formula.

$$Final\;Score=\frac{\sum_{i=1}^{n}\left(f_{i}\times w_{i}\right)}{N}$$

where

*f_i_* = frequency of responses at rank position *i*,
  *w_i_* = weight assigned to rank position *i*,
  *n* = total number of rank positions (number of interventions in the domain),
  *N* = number of interventions in the domain.

The bottom 30% of lowest-ranked interventions were removed between successive rounds.

### 2.5. Ethics

This study was performed with permission and approval from the Malawian College of Medicine Research and Ethics Committee (COMREC) P.11/21/3465, the Imperial College Research Ethics Committee (22IC7525), as well as the Directorate of Road Traffic and Safety Services at the Ministry of Transport, Malawi (C/RTSS/400/11/2/1).

## 3. Results

**Figure 1:**
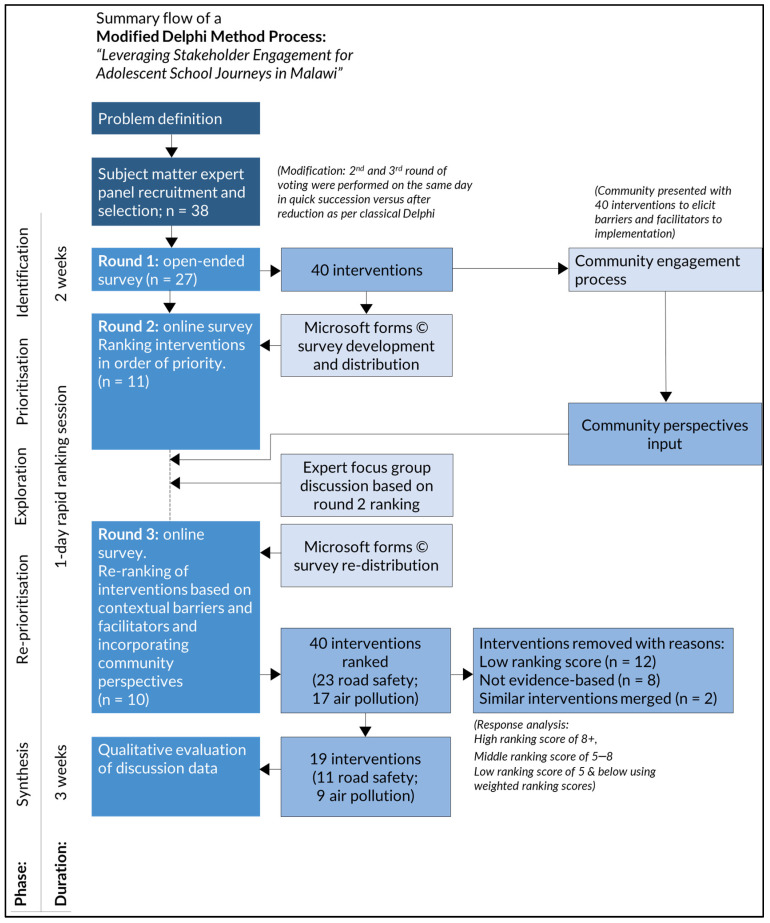
Conceptual diagram showing methodology of study, flow of respondents, and interventions along the process.

A total of 38 experts were invited to participate. These included 25 experts in road safety, 10 experts on air pollution and 3 with combined expertise. Of these, 27 (71.5%) completed Round 1, 11 completed Round 2, and 10 completed Round 3. Participant characteristics are shown in Table 2. The steps of this study are illustrated in the conceptual diagram (Figure 1).

### 3.1. Identification and Ranking of Interventions

In response to the Round 1 online survey, experts identified 40 distinct interventions in total, including 23 road safety interventions and 17 air pollution interventions.

**Figure 2:**
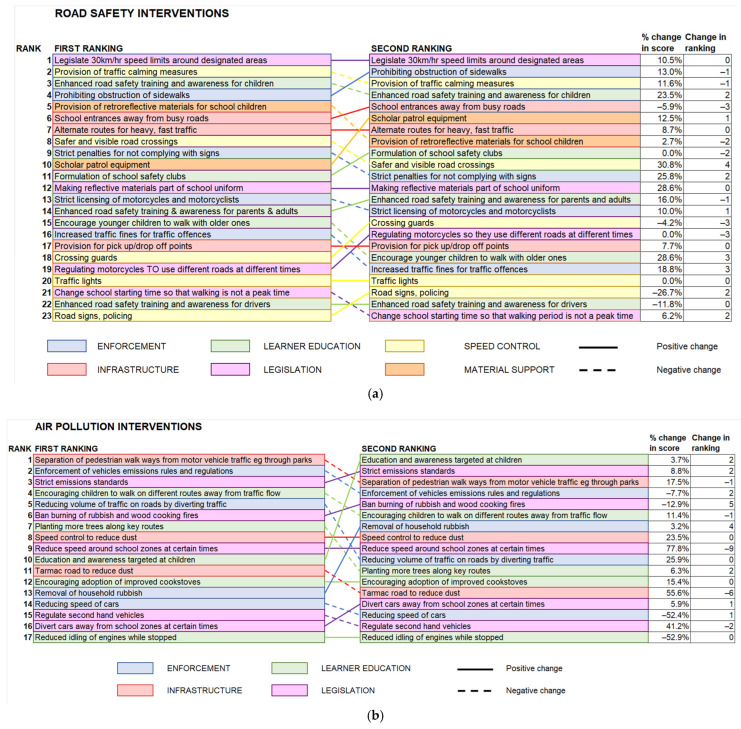
Ranking of interventions between 2nd round and 3rd round. (**a**) Road safety interventions; (**b**) air pollution interventions.

In Rounds 2 and 3, the interventions were ranked based on their priority scores. Round 3 included community perspectives. The ranking of interventions and the changes in ranking between rounds 2 and 3 are shown in Figure 2a,b.

### 3.2. Response Analysis and Reduction

**Figure 3:**
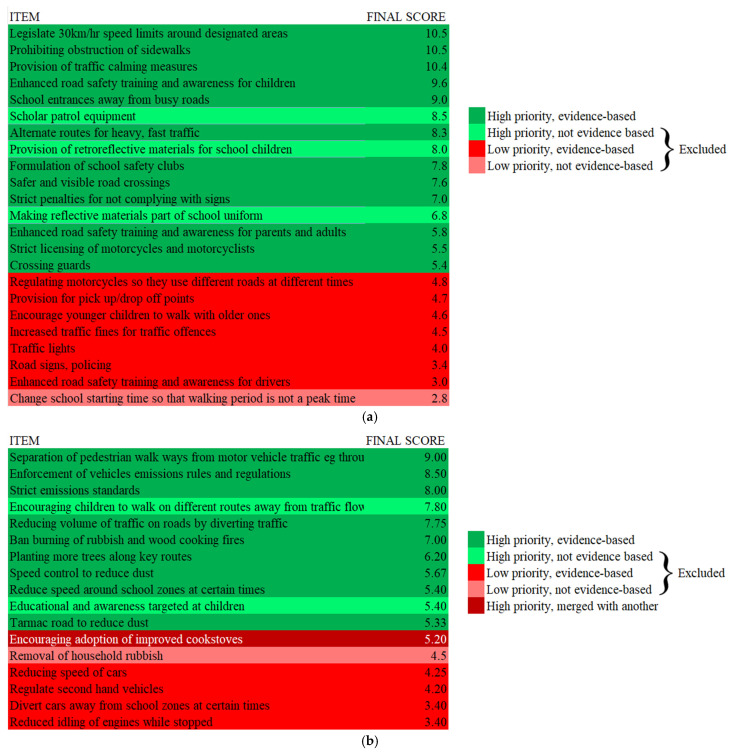
Delphi response analysis and removal of interventions. (**a**) Road safety; (**b**) air pollution.

Weighted ranking scores for each intervention were tabulated after Round 2. Interventions with low-ranking scores were removed from the lists, as shown in Figure 3a,b. Mapping of the identified interventions onto either the Save LIVES framework for road safety [12] or the modified Burns et al. framework for air pollution [15] is shown in Table 3 and Table 4. The road safety interventions identified by participants comprised 17 out of 22 Save LIVES interventions and four interventions without robust evidence as isolated interventions; three air pollution interventions were also not evidence-based and removed. In addition, two interventions with similar mechanisms of change were merged (Figure 1).

### 3.3. Community and Expert Perspectives on Barriers and Facilitators

Community members identified multiple barriers and facilitators to implementation of the identified interventions. There was a tendency among both the community members and experts alike to attribute road traffic crashes to errant behavior of children or drivers (recklessness, negligence, lack of knowledge, or excessive playfulness) more than the infrastructural, legislative, or situational environment. The most frequently identified road safety interventions by both experts and community members were related to more stringent enforcement of traffic rules and regulations and education or training of students, drivers, and parents. This appeared to be similar for air quality interventions, which were dominated by educative and enforcement interventions for vehicular sources and household sources, e.g., burning rubbish. Identified barriers for road enforcement interventions included the potential for corruption (both in issuing of licenses and collection of fines) and lack of sensitization or knowledge of road users on laws rendering them unenforceable. The idea of enforcement appeared to be mostly hindered by those factors. Some participants said the following:

*“I think it is impossible because some licenses are issued out illegally” (Participant A).*

*“Then it is impossible. This is because those entrusted with the responsibility of collecting the fines are corrupt so it would be a way of enriching themselves” (Participant B).*

*“People are buying licenses” (Participant C).*

*“Police officers …would demand money… That would not deter the would-be offenders” (Participant D).*

Also, it was suggested by some participants that road users don’t know about the road rules:

*“People have not yet been told about the offenses and penalties” (Participant E).*

Diversion of traffic through new routes or times was uniformly considered infeasible. Barriers to enforcement of roadside vendor cooking and burning included the potential to disempower those vendors and impact their income.

Regarding road safety, there appeared to be a reluctance to suggest infrastructural interventions because of the multiple identified financial barriers that were thought to be insurmountable. One community member is quoted as follows:

*“It is impossible because it is too expensive” (Participant C).*

There appeared to be a general sense of futility of suggesting infrastructural interventions due to political factors, cost, or lack of acceptability of interventions by the public.

On the implementation of slower speeds around schools, community members’ perspectives were that 30 km/h was too slow and disruptive to traffic flow in Blantyre. Participants suggest that lower speeds were

*“Impossible, 30 km/h is just a small speed limit” (Participant F),*

*“Would lead to congestion” (Participant A)*

*“Impossible, Blantyre was already given a small speed limit which is 60 km/h” (Participant G).*

The community perspective tended to focus on immediate, tangible barriers, such as the physical state of infrastructure (e.g., faded zebra crossings, lack of sidewalks), as well as an emphasis on the local socio-cultural context, such as corruption, lack of awareness, and practical daily challenges such as theft of signs. In contrast, the experts tended to identify more systemic and higher-level issues such as such as inadequate funding, legislative frameworks, and political commitment. They also tended to mention broader infrastructural and policy-related issues.

## 4. Discussion

This stakeholder engagement process set out to identify and seek consensus on interventions for enhancing road safety and reducing air pollution exposure among school-going children on their journeys to school in urban Blantyre, Malawi, with a strong emphasis on community perspectives and stakeholder engagement to ensure contextual appropriateness. Stakeholders prioritized speed limit enforcement, pedestrian infrastructure improvements, and emission controls, which aligned with relevant evidence-based frameworks while revealing an overemphasis on educational and enforcement measures despite limited evidence for effectiveness as standalone interventions. The findings from this mixed-methods approach uncover multiple important financial, political, and social barriers and facilitators to implementation of established road safety and air pollution interventions in urban Blantyre and give insights into the perception of the community and experts alike on the effectiveness of some interventions.

Odonkor et al. in Ghana categorized road safety challenges in Africa into institutional, executional, operational, behavioural, data-reporting, and financial dimensions [26]. The study revealed a preference among stakeholders for measures targeting driver and pedestrian behavior as well as some pessimism with respect to solvability of infrastructural and systemic issues. These attitudinal and institutional factors create implementation gaps similar to a case in Ghana from Odonkor et al. where the community often attributes crashes to individual behaviour rather than systemic factors, while simultaneously facing institutional barriers that hamper intervention efforts [26]. The perception of corruption as an impediment to enforcement measures emerged in both contexts, suggesting this might be a widespread challenge requiring specific attention in sub-Saharan Africa. Odonkor’s work reinforces this study’s observation that infrastructural interventions, though evidence-based, are frequently deprioritized due to perceived financial and political constraints. The insights gathered from this study underscore the benefits of engaging multiple stakeholders and the synergistic impact of addressing road safety and air pollution concurrently. The participatory approach brought forward a diversity of perspectives, thereby enabling the future implementation of a package of interventions to maximize contextual relevance, feasibility, and acceptability.

Road safety interventions identified and prioritized by both local and international stakeholders mapped relatively well onto the Save LIVES package. This is a WHO endorsed package of 22 evidence-based interventions for road safety, 17 of which were mapped onto the identified interventions, suggesting relative agreement with the evidence base. Respondents identified four interventions that were not part of the Save LIVES package, i.e., not evidence-based, and in particular overemphasized educational and awareness interventions that have been shown to have limited effectiveness on their own. The most frequently identified interventions related to education and awareness and stricter policing and enforcement. There was also an emphasis on enforcement and policing with regard to motorcycles, which appear to hold a particularly controversial place in public consciousness. Stakeholders did not recommend interventions related to post-crash response in their deliberations despite having clinicians in the group. Similarly, regarding air pollution interventions, those that related to education and awareness and banning of roadside cooking and burning suggested that local experts and community members reserved particular dislike for these roadside pollution sources. Published work from the same schools reveals that motorcycles are in fact not the fastest vehicles on roads within the school zone, (unpublished work). Poor road conditions, narrow sidewalk width, and quality of road crossings are all identified infrastructural gaps around the same schools [27]. The study findings are consistent with other studies from LMICs seeking consensus on road safety. Similar to a study from Ghana [28], this study found consensus on enforcement-based measures, including speed regulation, especially for commercial transport, as well as the need for driver training and road safety education. There was also agreement on the misunderstood role of powered two- and three-wheelers.

Conversations with the communities revealed a similar trend. Community members tended to attribute road crashes to human factors (dispositional and personality factors) while underemphasizing environmental, infrastructural, and systemic factors. Both groups appeared to attribute road crashes to recklessness, negligence, or lack of knowledge on the part of the drivers or excessive playfulness and lack of knowledge on the part of affected children. Thus, the most commonly identified interventions were more stringent enforcement and driver and student education.

On the other hand, infrastructural and legislative issues were underemphasized because of the perceived difficulty in implementing these interventions. This study reveals important considerations if enforcement and policing interventions are to be implemented in Blantyre. Increasingly stringent enforcement and policing carries the risk of encouraging corruption without discouraging unsafe road behaviors [29]. In addition, child road safety education as an isolated intervention has been implemented in some form or other in Malawi for at least a decade [30] and has had limited effectiveness in multiple African countries.

These obstacles have been identified in other countries and have been overcome in different ways. Rwanda’s experience using Automated Speed Enforcement systems led to a substantial monthly decrease in road traffic deaths and cuts out the human factor that might be susceptible to corruption [31]. In Tanzania, concerns about affordability of infrastructural road interventions were circumvented using low-cost improvements (the SARSAI model) and public-private partnerships that engaged local businesses to fund infrastructure improvements around schools through cost-sharing models [17]. In Ghana, corruption was addressed using community oversight mechanisms and targeted anti-corruption measures implemented alongside traffic enforcement efforts [26].

The study uncovered important equity issues with some interventions. For example, while bans on second-hand cars were suggested by international experts, the community and local experts pointed out that banning second-hand cars would disadvantage car ownership for those that cannot afford brand new cars. Importation of second-hand vehicles into Malawi was substantial until 2010 when the excise duty levied on older imported second-hand vehicles was increased, constituting an effective ban [32]. Making vehicles accessible to a wider proportion of the population was considered by community members to be a positive for poorer people. In addition, while banning roadside cooking and burning was identified with some frequency, it was recognized that this would impact the livelihoods of the road-side vendors who depended on the income from those activities. Equity concerns highlight unintended social and economic harms, such as restricting mobility through second-hand car bans or threatening livelihoods with roadside cooking restrictions. These must be deprioritized until mitigating measures can be applied simultaneously to protect the vulnerable.

This work shows the importance of multisectoral collaboration incorporating the community and experts but also including a wider range of stakeholders in the process of identifying barriers and facilitators and designing interventions [33]. Policymakers at all levels, including local authorities, provincial leaders and national government across line ministries (education, health, law enforcement, and public works), should also be included in these conversations [34]. However, because of the heterogeneity of these stakeholders, these may need to be separated to ameliorate power imbalances from inhibiting collaborative sharing.

There are well-described synergies between interventions aiming to improve road safety and those for reducing air pollution, particularly from vehicular sources (Figure S1). The relationship between vehicle speed and the reduction of road crashes is well described; however, the relationship with emissions is not as clear. Reduction in vehicle speed on highways does clearly reduce emissions; however, in city streets there may be no change or marginal increase, particularly after traffic calming measures, where there is a tendency for drivers to accelerate out of speed bumps or stop signs or idle at intersections. However, encouraging slow speed and steady traffic flow without stopping and starting does reduce emissions, for example, the use of traffic roundabouts and traffic circles rather than stop signs or traffic lights. Because of this, the intervention list includes anti-idling measures, no-stop traffic calming measures, and overall low school-zone speed limits to benefit from both the road safety and air quality benefits.

### Limitations

This modified Delphi study was limited by the small sample size that was obtained because of relatively low engagement despite persistent efforts to improve response rates. This study also had a limited pool of air pollution experts in Malawi, likely because of a dearth of local expertise in this area. In addition, there was a notable absence of policymakers among stakeholders consulted. This may have accounted for unrealistic expectations of legislative interventions as well as a general tendency toward pessimism with respect to systemwide policy and legislative actions displayed by local community members and experts alike. This mixed-method Delphi approach/qualitative study showed how qualitative rounds and discussion can augment modified Delphi studies where small sample sizes are anticipated. This study found that the interplay between expert discussion and incorporation of community inputs resulted in rich, deep discussions and uncovered important impediments to potential implementation of interventions in Malawi.

## 5. Conclusions

This study provides valuable insights into stakeholder-prioritized interventions for enhancing road safety and reducing air pollution exposure among school-going children in urban Blantyre. Through a modified Delphi approach, the research identified several key interventions, with speed limit enforcement, pedestrian infrastructure improvements, and emission controls receiving highest priority. The findings revealed important contextual barriers to implementation, including financial constraints, corruption concerns, and systemic challenges within Malawi’s institutional framework.

The research underscores the benefit of integrating both expert knowledge and community perspectives to produce context-sensitive solutions. While experts emphasized systemic and legislative approaches, community members highlighted immediate tangential concerns and practical implementation challenges. This tension between ideal interventions and practical realities illustrates the complex landscape of public health implementation in resource-constrained settings.

The synergies between road safety and air pollution interventions identified in this work offer promising avenues for integrated approaches. Interventions such as speed reduction measures, traffic flow optimization, and vehicle emission controls can potentially address both health concerns simultaneously, maximizing impact with limited resources.

As next steps, a policy dialogue with government stakeholders has been initiated to translate these findings into actionable policies (known as “REMPolicy”). This model of multistakeholder engagement provides a replicable framework for other low- and middle-income countries facing similar public health challenges.

Future work should focus on evaluating the effectiveness of the proposed interventions, exploring potential avenues for scaling up, and understanding the mechanisms that can help sustain these interventions over time.

## Figures and Tables

**Table 1:** Eligibility criteria for Delphi experts.

Type of Participant	Criteria for Eligibility
Academic researcher	Lead author or co-author in at least one article pertaining to either road safety or air pollution in Malawi published after 2010.
Clinicians	Medically qualified clinicians with at least 2 years’ experience in the field of emergency medicine, trauma, respiratory diseases, or pediatrics.
Non-Governmental Organizations and policymakers	At least 6 months’ experience in the arena of road safety, environmental health, or child health.

**Table 2:** Characteristics of respondents.

	Invitations (n = 38)	Round 1 (n = 27)	Round 2 (n = 11)	Round 3 (n = 10)
Gender				
Male	22	14	4	4
Female	15	12	7	6
Prefer not to say	1	1	0	0
Country of residence				
Local	21	12	5	4
International	17	15	6	6
Current Role				
Academia	11	11	4	4
NGO	6	6	3	3
Multi-lateral organisation	2	1	0	0
Government	11	5	2	1
Clinical	8	4	2	2
Domain of expertise				
Road Safety	25	17	11	10
Air pollution	10	7	0	0
Combination	3	3	0	0

**Table 3:** Framework for analysis of identified road safety interventions used in the study.

**Save LIVES Component**	**Interventions Identified by Experts**	Code Frequency *
Speed Management	Provision of traffic calming measures	8
	Legislate 30 km/h speed limits around designated areas	3
Leadership on road safety	Enhanced road safety training and awareness for children	6
	Enhanced road safety training and awareness for drivers	4
	Enhanced road safety training and awareness for parents and adults	2
	Formulation of school safety clubs	1
	Crossing guards	1
	Encourage younger children to walk with older ones	1
Infrastructure design and improvement	Safer and visible road crossings	5
	Alternate routes for heavy, fast traffic	2
	School entrances away from busy roads	1
	Provision for pick up/drop off points	1
Vehicle safety	-	0
Enforcement of traffic laws	Strict licensing of motorcycles and motorcyclists	4
	Strict penalties for not complying with signs	3
	Increased traffic fines for traffic offences	3
	Regulating motorcycles so they use different roads at different times	3
	Road signs, policing	3
Survival	-	0
Non-evidence-based interventions	Making reflective materials part of school uniform	2
	Provision of scholar patrol equipment	1
	Change school starting time so that walking period is not a peak time	1
	Provision of retroreflective materials for school children	1

* Code frequencies show how many times the intervention was mentioned by a participant.

**Table 4:** Framework for analysis of identified air pollution interventions used in the study.

Framework Component	Interventions Identified by Experts	Code Frequency *
Residential sources	Ban burning of rubbish and wood cooking fires	3
	Encouraging adoption of improved cookstoves	1
Industrial sources	-	0
Vehicular sources	Reducing volume of traffic on roads by diverting traffic	4
	Enforcement of vehicles emissions rules and regulations	4
	Separation of pedestrian walkways from motor vehicle traffic, e.g., through parks	3
	Regulate second-hand vehicles	3
	Speed control to reduce dust	3
	Tarmac road to reduce dust	3
	Divert cars away from school zones at certain times	2
	Reduce speed around school zones at certain times	1
	Reducing speed of cars	1
	Strict emissions standards	1
	Reduced idling of engines while stopped	1
Miscellaneous sources	Planting more trees along key routes	1
Limited-evidence based (as isolated interventions)	Education and awareness targeted at children	5
	Removal of household rubbish	3
	Encouraging children to walk on different routes away from traffic flow	1

* Code frequencies show how many times the intervention was mentioned by a participant.

## Data Availability

The data presented in this study are available on request from the corresponding author due to privacy concerns of participants because of the low number of participants and the specific unique perspectives which may help unmask anonymous participants.

## Supplementary Materials

The following supporting information can be downloaded at https://www.mdpi.com/article/10.3390/ijerph22050758/s1: Table S1: Delphi Survey Questions Round 1, Table S2: Delphi Survey Questions Round 2, Table S3: Delphi Survey Questions Round 3, Table S4: Expert Focus Group Discussion Guide, Table S5: Community Focus Group Discussion Guide—teachers and parents, Figure S1: Synergies between road safety interventions and air pollution interventions.
