# Supplementary material for: Leveraging Stakeholder Engagement for Adolescent School Journeys in Malawi: An Exploration of Road Safety and Air Pollution Interventions
Source: Int J Environ Res Public Health. 2025 May 12;22(5):758. doi: 10.3390/ijerph22050758 (PMC12110807; doi:10.3390/ijerph22050758)
Supplement: Supplementary file 1 [file ijerph-22-00758-s001.zip › ijerph-3480324-supplementary.pdf]

Table S1

Delphi Survey Questions Round 1

Round 1: Identification of Potential Interventions (exported from web survey)

Demographics

1. What is your gender? [Male/Female/Prefer not to say]
2. What is your country of residence? [Open-ended]
3. Which of the following best describes your current professional role?
   - Academia
   - Non-Governmental Organization
   - Multi-lateral Organization
   - Government
   - Clinical practice
   - Other (please specify): _______
4. Which of the following best describes your domain of expertise?
   - o Road safety
   - o Air pollution
   - o Both road safety and air pollution

Road Safety Interventions

5. Please identify potential interventions that could improve road safety for school-going children on their journey to school in urban Blantyre, Malawi. List as many as you consider relevant. [Open-ended text boxes for up to 5 interventions]
6. For each intervention you have identified above, please:
   a) Describe the specific barriers that might prevent successful implementation of this intervention in urban Blantyre. [Open-ended]
   b) Describe specific facilitators that could support successful implementation of this intervention in urban Blantyre. [Open-ended]

Air Pollution Interventions

7. Please identify potential interventions that could reduce air pollution exposure for school-going children on their journey to school in urban Blantyre, Malawi. List as many as you consider relevant. [Open-ended text boxes for up to 5 interventions]
8. For each intervention you have identified above, please:
   a) Describe the specific barriers that might prevent successful implementation of this intervention in urban Blantyre. [Open-ended]

b) Describe specific facilitators that could support successful implementation of this intervention in urban Blantyre. [Open-ended]

Table S2

Delphi Survey Questions Round 2

Round 2: Ranking of interventions identified from round 1 (exported from web survey)

Road safety interventions prioritization

1. Below is a list of road safety interventions identified in Round 1. Please rank these interventions within each category according to their priority for implementation in urban Blantyre (1 = highest priority/rank).

Enforcement:

- Prohibiting obstruction of sidewalks
- Strict penalties for not complying with signs
- Strict licensing of motorcycles and motorcyclists
- Increased traffic fines for traffic offences

Road safety infrastructure:

- Alternate routes for heavy, fast traffic
- School entrances away from busy roads
- Provision for pick up/drop off points

Learners' education

- Enhanced road safety training and awareness for children
- Encourage younger children to walk with older ones
- Formulation of school safety clubs
- Enhanced road safety training and awareness for parents and adults
- Enhanced road safety training and awareness for drivers

Legislation and policy:

- Legislate 30km/hr speed limits around designated areas
- Making reflective materials part of school uniform
- Regulating motorcycles so they use different roads at different times
- Change school starting time so that walking period is not a peak time

Material support:

- Provision of retroreflective materials for school children
- Scholar patrol equipment

Speed control

- Provision of traffic calming measures
- Safer and visible road crossings
- Traffic lights
- Crossing guards
- Road signs, policing

Are there any additional interventions that you believe should be included that haven't been shown above?

Air pollution interventions prioritization

2. Below is a list of air pollution interventions identified in Round 1. Please rank these interventions within each category according to their priority for implementation in urban Blantyre (1 = highest priority/rank).

Enforcement:

- Enforcement of vehicles emissions rules and regulations
- Reducing volume of traffic on roads by diverting traffic
- Removal of household rubbish
- Reducing speed of cars

Infrastructural changes:

- Separation of pedestrian walkways from motor vehicle traffic eg through parks
- Speed control to reduce dust
- Tarmac road to reduce dust

Legislation/policy:

- Strict emissions standards
- Ban burning of rubbish and wood cooking fires
- Reduce speed around school zones at certain times
- Divert cars away from school zones at certain times
- Regulate second hand vehicles

Public awareness/Education/Behaviour change:

- Educational and awareness targeted at children

- Planting more trees along key routes
- Encouraging children to walk on different routes away from traffic flow
- Encouraging adoption of improved cookstoves
- Reduced idling of engines while stopped

Are there any additional interventions that you believe should be included that haven't been shown above?

Table S3

Delphi Survey Questions Round 3

Round 3: Re-ranking of interventions – same day expert survey after round 2 (exported from web survey)

Please re-rank the interventions based on our discussion and community perspectives:

Road Safety Interventions Re-ranking

1. Below is a list of road safety interventions. Please re-rank these interventions within each category according to their priority for implementation in urban Blantyre, considering both the expert discussion and community perspectives (1 = highest priority).

Enforcement:

- [list of interventions with low priority interventions removed]

Road safety infrastructure:

- [list of interventions with low priority interventions removed]

Learners' education

- [list of interventions with low priority interventions removed]

Legislation and policy:

- [list of interventions with low priority interventions removed]

Material support:

- [list of interventions with low priority interventions removed]

Speed control

- [list of interventions with low priority interventions removed]

Air pollution interventions prioritization

2. Below is a list of road safety interventions. Please re-rank these interventions within each category according to their priority for implementation in urban Blantyre, considering both the expert discussion and community perspectives (1 = highest priority).

Enforcement:

- [list of interventions with low priority interventions removed]

Infrastructural changes:

- [list of interventions with low priority interventions removed]

Legislation/policy:

- [list of interventions with low priority interventions removed]

Public awareness/Education/Behaviour change:

- [list of interventions with low priority interventions removed]

Table S4

Expert Focus Group Discussion Guide

Risk Elimination on Walks to School - 2nd Round Delphi Consultation

Introduction (15 minutes) via powerpoint

- Welcome and thank you for participating in this focus group discussion
- Brief overview of the REmWalks project
- Presentation of Round 2 rankings and explanation of how prioritization was calculated
- Explanation of the discussion format and objectives

Discussion of Road Safety Intervention Priorities (45 minutes)

For each intervention:

- What are the main barriers to implementing these measures in the local context? Why?
- How can these barriers be addressed?
- What facilitators could help overcome these barriers? Why?
- Under what circumstances would this intervention be feasible?

Additional questions

- What factors influenced your ranking decisions?
- Were there any interventions you felt strongly about prioritizing? Why?
- Were there any interventions you felt should not be prioritized? Why?
- How do community perspectives align or differ from your expert assessment?
- How might these perspectives influence implementation strategies?
- Are there community-identified barriers or facilitators that changed your thinking about priorities?
- What mechanisms could increase transparency in the enforcement process?
- How could enforcement be implemented in a way that minimizes corruption?
- Why do you think this intervention was highly prioritized despite limited evidence for effectiveness as an isolated intervention?

Table S5

Risk Elimination on Walks to School
Community Focus Group Discussion Guide – teachers and parents

Objective: To gather stakeholder perspectives on the feasibility, acceptability, and perceived impact of proposed interventions related to road safety and air pollution affecting learners.

Instructions for the Facilitator:
For each set of proposed interventions, repeat the full set of questions below. If participants express a preference, focus discussions on individual interventions within a category. Ensure that both positive and negative perspectives are explored. Encourage participants to provide real-life examples and practical insights.

Discussion Questions (to be used for each intervention or intervention set):

- What is your opinion on this set of interventions?
- Do you think these interventions would be useful in addressing the road safety/air pollution challenges that learners face on their way to school? Why?
- What are your thoughts on the acceptability of the interventions among parents, teachers and the learners?
- What could be the challenges or limitations associated with implementing these interventions?
- Are there any known practices or policies that may conflict with the interventions?
- What is your opinion on the communities' (parents, teachers, learners) participation in implementing these interventions?
- What could be done to ensure they are effectively implemented?

Figure S6

Supplementary figure 1. Synergies between road safety interventions and air pollution interventions.

Intervention	Road Safety	Air Pollution
Speed Management		
30 km/h zones	Reduce the risk of injury and death of vehicle occupants	Reduce carbon dioxide, nitrous oxide and particulate matter emissions
Speed bumps	Reduce speed and crash frequency, injuries and fatalities	Increase emissions from stop-start driving
Road narrowing, and mini-roundabouts ("no-stop" infrastructure)	Reduce vehicle speeds, and reduce crashes and injury severity	Reduce carbon dioxide emmisions
Legislative interventions		

Emission zones	Decreasing overall traffic density and vehicle-pedestrian conflict	Reduce vehicle emmisions in target area
Green infrastructure Barriers	Physical separation reduces vehicle-pedestrian conflicts	Reduction in pedestrian exposure to PM2.5
Route Optimization/Diversion		
Pedestrian separation/walkways	Reduction in school zone pedestrian-vehicle conflicts	Reduction in traffic associated pollutants and emissions.
School entrances away from busy roads	Reduces student exposure to crashes	Reduces student exposure to emissions
Mode shift to active transportation	Reduced vehicle volumes lowers crash probability	reduction in transport-related emissions
Traffic calming PLUS anti-idling laws	Reduce road crashes, injuries and mortality as well as emmisions	
